# Increasing Cytomegalovirus Detection Rate from Respiratory Tract Specimens by a New Laboratory-Developed Automated Molecular Diagnostic Test

**DOI:** 10.3390/microorganisms8071063

**Published:** 2020-07-16

**Authors:** Huey-Pin Tsai, Chun-Sheng Yeh, I-Ting Lin, Wen-Chien Ko, Jen-Ren Wang

**Affiliations:** 1 Department of Pathology, National Cheng Kung University Hospital, College of Medicine, National Cheng Kung University, Tainan 704, Taiwan; yeh30562@gmail.com (C.-S.Y.); yvonne.lin8047@gmail.com (I.-T.L.); 2 Department of Medical Laboratory Science and Biotechnology, College of Medicine, National Cheng Kung University, Tainan 701, Taiwan; 3 Division of Infectious Diseases, Department of Internal Medicine, National Cheng Kung University Hospital, College of Medicine, National Cheng Kung University, Tainan 704, Taiwan; winston3415@gmail.com; 4 Department of Internal Medicine, College of Medicine, National Cheng Kung University, Tainan 701, Taiwan; 5 Center of Infectious Disease and Signaling Research, National Cheng Kung University, Tainan 701, Taiwan; 6 National Institute of Infectious Diseases and Vaccinology, National Health Research Institutes, Tainan 704, Taiwan

**Keywords:** cytomegalovirus, respiratory tract specimens, automated molecular diagnostic test

## Abstract

Lots of automated molecular methods for detecting cytomegalovirus (CMV) DNA in the blood are available, but seldom for various clinical specimens. This study was designed to establish a highly sensitive automated assay to detect CMV DNA in non-blood specimens. We designed a new QMT assay using QIAGEN artus CMV RG polymerase chain reaction (**Q**-CMV PCR) kit applied on the BD **M**AX system and compared with the other assays, including an RGQ assay (LabTurbo auto-extraction combined Q-CMV PCR kit on **R**otor-**G**ene-**Q** instrument), and in-house PCR assay. A total of 1067 various clinical samples, including 426 plasma, 293 respiratory tract specimens (RTS), 127 stool, 101 cerebral spinal fluid, 90 vitreous humours were analysed. Examining CMV DNA in simultaneous specimens of the same immunocompromised patient with respiratory symptoms, the detection rate of RTS (93.6%, 88/94) was significant higher than plasma (65.9%, 62/94). The positive rates for plasma samples with a low CMV viral load (<137 IU/mL) and diagnostic sensitivity of QMT, RGQ, and in-house assays were 65% and 99.1%, 45% and 100%, 5% and 65.5%, respectively. The QMT assay performs better, with shorter operational and turnaround time than the other assays, enabling the effective and early detection of CMV infection in various clinical specimens, particularly for RTS.

## 1. Introduction

Cytomegalovirus (CMV), a common human pathogen, can affect different organs, especially in immunocompromised patients [1,2]. The quantitation of CMV particles in the blood for pre-emptive and effective therapy is the treatment guideline to reduce morbidity and mortality [3,4,5,6]. However, CMV reactivation patients with non-DNAemia stage will delay the timing of medical intervention, especially in organ-related diseases such as pneumonia [7], retinitis [8], colitis [9] and inflammatory mammary gland [10]. Therefore, the accurate detection of CMV particles in various specimens is critical for clinical decision-making and prescribing appropriate treatment regimens.

The polymerase chain reaction (PCR) approach is widely used for rapid diagnosis of CMV infection [11,12,13,14]. However, commercial automated extraction combined with real-time PCR platforms (In Vitro Diagnostics, IVD), such as Roche Cobas AmpliPrep/TaqMan 48, Abbott m2000SP/2000RT and QIAGEN QIAsymphony/Rotor Gene-Q, have only been validated for the detection of CMV DNA in blood samples [15,16,17]. Few assays for non-blood specimens have been validated and reported [18,19]. Considering the matrix complexity of non-blood specimens, the performance of CMV PCR testing of such specimens should be validated to ensure the quality of assays.

Although a manual PCR approach for the detection of viral nucleic acids has gradually been replaced with highly sensitive and straightforward automated platforms, most of these platforms require specific infrastructure, equipment, and trained staff. A fully integrated, automated platform for nucleic acid extraction and real-time PCR using the Becton Dickinson (BD) MAX system enables many commercial IVD assays for infectious agents [20,21,22,23,24,25,26], with the possibility of creating user-defined protocols using open-system reagents to meet emerging diagnostic demands and address regional healthcare needs. The evaluation of laboratory developed assays using the BD MAX system for the detection of numerous microbes has recently been published, however, none of them pertain to CMV [27,28,29,30,31,32]. Herein, we designed a new QMT assay applied on the BD MAX system for the detection of CMV DNA in various clinical specimens, especially different respiratory tract specimens. In this study, we validated the performance of the QMT assay. In addition, the diagnostic performance and workflow of QMT assay, commercially available CMV assays and an in-house CMV PCR assay were compared.

## 2. Materials and Methods

### 2.1. Clinical Samples and Ethics Statement

A total of 1067 various clinical samples including 426 plasma, 293 respiratory tract specimens (RTS), 127 stool, 101 cerebral spinal fluid, 90 vitreous humour, 18 urine, 5 breast milk, 5 bone marrow and 2 lung tissue, suspected of being infected with CMV and collected at the Clinical Virology Laboratory of National Cheng Kung University Hospital (NCKUH), were analyzed retrospectively. Sample aliquots (stored at −70 °C) were simultaneously tested by the specified assays. The Institutional Review Board of NCKUH with a number of B-ER-106-316 approved the study. The demographic and clinical information for the patients was unlinked prior to analysis, and hence the patients’ informed consent was waived.

### 2.2. QMT Assay: QIAGEN Artus CMV RG PCR (Q-CMV PCR) Reagents Applied on BD MAX

The BD MAX system allows nucleic acid extraction and real-time PCR using a single instrument. We designed the following protocols for the new QMT assay. Sample preparation for BD MAX: first, 15 µL of internal positive control (IPC) from the artus CMV RG PCR (Q-CMV PCR) kit (QIAGEN GmbH, GERMANY) was added to each sample-processing tube of the ExK DNA-1 extraction kit (DNA-1 kit, ref: 442818; Becton Dickinson, Breda, the Netherlands). Next, 200 µL of liquid specimens was added to SPT, while 2 g of solid stool or 10 µL of liquid stool were used. Liquefy the viscous sputum or BAL: an equal volume of viral transport medium consisting of Eagle’s Minimum Essential Medium (EMEM), penicillin/streptomycin, and 0.5% gelatine was added to viscous respiratory specimens, which were then vortex-mixed for over 3 min. Five new designed different BD MAX extraction programmes were used for the different specimens (Table 1).

The new reaction conditions of the Q-CMV PCR kit protocol on BD MAX realtime PCR instrument were modified as follows: 12.5 µL of DNA with 12.5 µL PCR master mix in each PCR reaction. Fluorescent signal gain for Cycling Green and Cycling Yellow was used for detecting CMV and IPC, respectively.

### 2.3. Validation of QMT Assay

Assay accuracy was determined using external quality control (College of American Pathologists Proficiency Testing (CAP-PT)) specimens. Precision was evaluated by testing the aliquots from pooled positive plasma samples and comparing the results from intra-runs and inter-runs. Clinical specimens containing herpes simplex virus, varicella-zoster virus, Epstein–Barr virus, hepatitis B virus, hepatitis C virus, influenza A and B virus, BK virus and enterovirus, were tested for analytical specificity.

### 2.4. RGQ Assay and in-House PCR Assay

The automated LabTurbo 48 Compact extraction system (Taigen Bioscience Corp., Taipei, Taiwan) with LabTurbo virus mini kit (cat. no. LVN480-300) were used for DNA extraction for RGQ and in-house PCR assays. RGQ assay using the QAIGEN artus CMV RG PCR (Q-CMV PCR) kit constituting a ready-to-use reagent for the detection of CMV DNA was validated for the Rotor-Gene-Q real-time PCR instrument, as suggested by the manufacturer. The in-house PCR assay was performed as previously described [33].

### 2.5. Roche Cobas CAP/CTM CMV Real-Time PCR Assay (Roche Assay)

The Roche CMV assay relies on the Cobas AmpliPrep/Cobas^®^ TaqMan system, which consists of a Cobas AmpliPrep system for automatic nucleic acid isolation with magnetic beads and Cobas^®^ TaqMan system for real-time PCR (Roche Molecular Diagnostics, Pleasanton, CA, USA). In the current study, the Roche assay was only used for the analysis of plasma specimens, as per manufacturer’s recommendations.

### 2.6. Comparison of QMT Assay to the Others Assays

**Figure 1:**
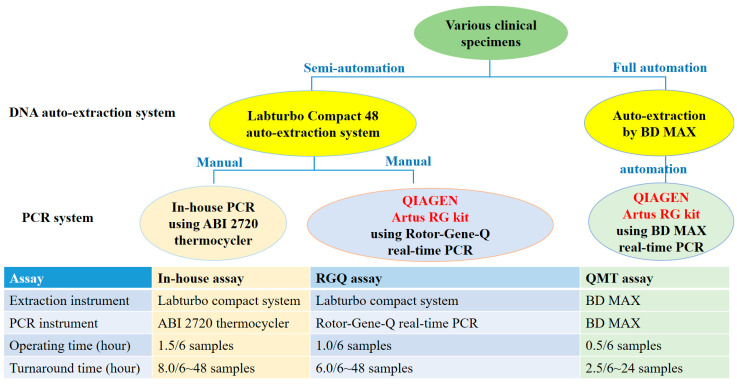
Comparative workflows for the in-house, RGQ, and QMT assays for detection of cytomegalovirus (CMV) nucleic acid in various clinical specimens. Maximum 24 samples for QMT assay vs. 48 samples for the others two assays in one batch.

Diagnostic sensitivity and specificity were evaluated by comparing the performance of different CMV PCR assays with clinical specimens. A total of 205 various clinical specimens were simultaneously used for the evaluation of diagnostic performance of the in-house, RGQ, and QMT assays (as specified below). The workflow is shown in Figure 1.

### 2.7. Statistical Analysis

Post hoc statistical analysis was used for the performance of QMT assay with various clinical specimens. The statistical analysis of CMV DNA detection rate in respiratory tract specimens and plasma of QMT assay was performed using Fisher’s exact test. The limit of detection (LOD) for the MAX assay was determined by analyzing dilutions of pooled CMV-positive plasma samples (quantified using the Roche CMV assay) in replicates of eight on three separate days. Assay LOD was determined by using Probit analysis (SPSS Statistics for Windows, v. 17.0., SPSS Inc., Chicago, IL, USA) with 95% probability within a 95% confidence interval (95% CI). Regression analysis correlating the different assays and agreement between viral loads was performed using the Bland–Altman method with MEDCALC^®^ statistical software (https://www.medcalc.org/, accessed on 15 July 2020). Data were log_10_-transformed prior to analysis.

## 3. Results

### 3.1. The Performance of QMT Assay in Various Clinical Specimens

A total of 1067 various clinical specimens were simultaneously tested and analyzed using QMT assay (Table 2). Most of positive CMV detection rate was found in the specimens of plasma (110/294, 37.4%) and BAL (80/294, 27.2%) samples (*p* < 0.05). In addition, CMV detection rate in the respiratory tract specimens was found to be higher (127/293, 43.3%) than that in plasma specimens (110/426, 25.8%). Furthermore, no amplification curve for the internal control was noted for 78 specimens (78/1067, 7.3%) with a significantly higher invalid rate of IPC in the bone marrow (4/5, 80%) and stool specimens (32/127, 25.2%) (*p* < 0.05).

### 3.2. CMV Nucleic Acid Detected in Simultaneous Specimens of Respiratory Tract and Plasma in Patient with Respiratory Symptoms by QMT Assay

In order to confirm the detection incidence of plasma and respiratory tract specimens by QMT assay, we analysed the detection result of simultaneous specimens from the same patient (Table 3). There were 94 episodes (91 patients) with various co-morbidity diseases including transplant, solid organ and hematology malignancy, immunodeficiency containing HIV, TB and SLE, diabetes mellitus, hypertension and cardiovascular disease. Fifty-nine episodes with CMV DNA detected in both plasma and respiratory tract specimens. However, 35 episodes had only one specimen with detected CMV DNA, 32 detected in respiratory tract specimens only and three were detected in plasma only respectively. Examining CMV DNA in simultaneous specimens of the same patient with respiratory symptom, we found the detection rate of respiratory tract specimens was significantly higher than plasma (*p* < 0.05) either in individual co-morbidity disease group or in all patients by QMT assay (Table 2).

### 3.3. Accuracy and Precision of QMT Assay

The accuracy of QMT assay was tested using CAP-PT external quality control specimens (Table 4). Comparison of new QMT assay, Roche assay and in-house assays revealed complete agreement in the results for qualitative and quantitative analyses. Variation in the viral load determined by the QMT and Roche assays was below 0.5 log_10_ international units (IU)/mL, except for sample 17-VLS-13 (10-fold diluted, owing to the limited volume of retrospective CAP-PT specimens). Further, a strong correlation was observed between QMT and Roche assays by regression analysis (R^2^ = 0.835). The coefficients of variation (CV%) ranged from 0.43–1.06% (data not shown), demonstrating the high precision of the QMT assay.

### 3.4. Analytical Sensitivity and Specificity of QMT Assay

Positive pooled plasma samples were simultaneously quantitatively analyzed via Roche assay (IU/mL) and QMT assay (copy/mL); 1 copy/mL of QMT assay corresponds to 0.16 IU/mL (data not shown). The analytical sensitivity of QMT assay was determined by analyzing serial dilutions of highly positive pooled plasma samples in CMV-negative normal human plasma. The positive rate in samples containing 2.1, 2.0, and 1.7 log_10_ copies/mL in replicates of ten was 90%, 80% and 50%, respectively (data not shown). According to Probit analysis, a QMT assay LOD for CMV DNA detection in 95% of replicates in plasma samples was 82 IU/mL (95% CI, 57~100). The QMT assay showed no cross-reactivity with other tested viruses (data not shown).

### 3.5. Comparison of the Performance Between In-House, RGQ, and QMT Assays Using Frozen Residual Plasma Specimens Positive in Roche Assay

The performance of in-house, RGQ, and QMT assays was tested using frozen residual positive plasma specimens previously analysed by the Roche assay (Table 5). Twenty Roche-assay-positive plasma specimens containing over 137 IU/mL (LOD of Roche assay) were all tested positive by RGQ and QMT assays. However, for 40 Roche assay positive plasma specimens with a low CMV viral load (<137 IU/mL), the positivity rate for the in-house, RGQ, and QMT assays was 5% (2/40), 45% (18/40), and 65% (26/40), respectively.

### 3.6. Diagnostic Performance of the In-House, RGQ, and QMT Assays with Various Clinical Specimens

To determine diagnostic performance, 205 clinical specimens were simultaneously evaluated by the in-house, RGQ, and QMT assays. The diagnostic sensitivities of the assays were 65.5%, 100% and 99.1%, respectively, whereas the diagnostic specificities were 100%, 87.3% and 91.5%, respectively (Table 6).

### 3.7. Quantitative Analysis of Clinical Specimens by Using RGQ and QMT Assays

**Figure 2:**
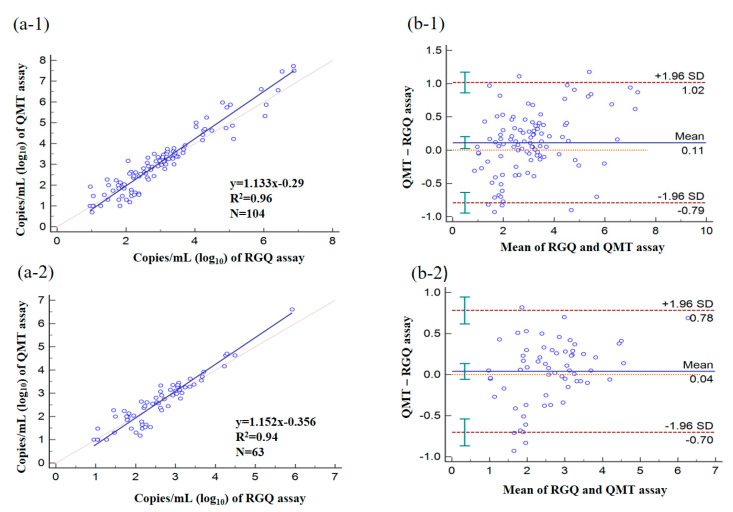
Quantitative analysis of clinical specimens using RGQ and QMT assays. Deming regression and Pearson correlation (**a**), and Bland–Altman analysis (**b**) of the RGQ and QMT assays. CMV DNA load (log_10_ IU/mL) determined for positive samples among all tested specimens (−1, *n* = 104) and plasma specimens (−2, *n* = 63) tested by both assays.

As shown in Figure 2, the viral loads in clinical specimens were determined as CMV-positive by using RGQ and QMT assays were significantly correlated. The Pearson correlation coefficient for the two assays was 0.96 for all specimens tested Figure 2(a-1) and 0.94 for plasma specimens Figure 2(a-2). According to the Bland–Altman analysis, viral load values determined using QMT assay were, on average, 0.11 log_10_ copies/mL in all tested specimens Figure 2(b-1) and 0.04 log_10_ copies/mL in plasma specimens Figure 2(b-2) higher than those determined using RGQ assay (*p* < 0.05).

### 3.8. Comparison of Workflows for the Detection of CMV Nucleic Acid in Various Clinical Specimens

The operating time as well as the turnaround time for CMV DNA detection by the in-house, RGQ, and QMT assays were compared (Figure 1). The turnaround time of QMT assay was less than that of the other two assays in one batch. Further, the shorter operating time and simpler steps of QMT assay proved to be more convenient for the user than the other two assays.

## 4. Discussion

The accurate and rapid detection of CMV in various specimens is essential for the timely treatment of CMV-related disease. Here, we present a user-defined protocol using the fully automated BD MAX system for CMV detection. In the current study, we have successfully devised CMV nucleic acid extraction protocols for the BD MAX instrument for multiple non-blood samples (Table 1). Although EDTA plasma was the most suitable sample for the artus CMV RG PCR kit as recommended by the manufacturer, we used this kit on BD MAX real-time PCR system with excellent test accuracy, precision, sensitivity, and specificity.

CMV is often found in various clinical specimens; however, blood is the only specimen that had been validated for use in these tests to date, save for the fully automated ELITe platform (https://www.elitechgroup.com/product/cmv-elite-mgb-kit-ingenius, accessed on 15 July 2020), which has also been validated for non-blood specimens such as amniotic fluid, urine, CSF, and saliva. In our developed protocols, we had validated additional clinical specimen types (Table 2) including respiratory tract specimens, stool, cerebral spinal fluid, vitreous humour, urine, breast milk, bone marrow and lung tissues using QMT assay.

According to a recent study, LOD for the commercial CMV DNA standard, detected using Roche CAP/CTM CMV platform in BAL, is lower than that in CSF and urine [18]. Costa et al. (28th European Congress of Clinical Microbiology and Infectious Disease in 2018, abstract P0496) reported an LOD for CMV ELITe platform of 57 IU/mL for amniotic fluid, 151 IU/mL for urine, and 44 IU/mL for saliva. Consequently, LOD values for CMV detection in various clinical specimens by using different methods vary greatly. The LOD of QMT assay developed in this study (82 IU/mL) was lower than that of the Roche assay (<137 IU/mL) in plasma specimens. Hence, the LOD for QMT assay in other types of clinical specimens should be investigated further.

Precise detection of CMV in specimens from immunocompromised patients with clinical manifestations of disease is crucial for therapeutic regimes. However, the ability of various commercial kits to manually extract CMV DNA from spiked human specimens varies [34]. Further, compared to other clinical specimens, respiratory tract specimens have been reported to account for the highest CMV PCR detection rate [18,19,35], for which similar results were found in this study (Table 2 and Table 3). Comparison of the sensitivities of different extraction protocols in various clinical specimens for CMV PCR requires further investigation.

Further, we observed an abnormally high IPC invalid rate for MAX assay with bone marrow 80% (4/5) and stool specimens 25.2% (32/127) (Table 2). Bone marrow, with high concentrated haemoglobin, yields high invalid PCR results and a high IC invalid rate in stools may be due to the presence of PCR inhibitors, including complex polysaccharides, and lipids [36,37]. The manufacturer has validated the BD MAX DNA-1 kit, used in this study, for the extraction of DNA from plasma, serum, or neat urine specimens, however, not from stool and bone marrow. Therefore, the BD MAX DNA-4 kit (validated for stool by the manufacturer) was tested to resolve the issue of IPC invalidity of the DNA-1 kit. Indeed, the invalid rate of IPC DNA in this study decreased to 9.5% (2/21) by the DNA-4 kit. Using an appropriate DNA extraction kit may serve to reduce the invalid rate in various clinical specimens.

Previous studies [16,38] reported a difference within 0.1 log_10_ copies/mL of the mean CMV viral load for plasma samples analysed using two different automated or semi-automated extraction instruments in conjunction with the same PCR reagents. However, the differences between two automated systems exceeded 0.5 log_10_ copies/mL when whole-blood or plasma specimens were tested [15,17,39]. Of note, the current study is the first to compare the quantitation of CMV DNA in various clinical specimens by two automated extraction and detection systems.

Finally, the fully automated BD MAX assay entails less manual intervention than the semi-automated RGQ assay, especially during PCR reaction preparation. Hence, the QMT assay, with a short operating time, is more convenient than RGQ assay for the analysis of small batches of specimens (six or less) in the laboratory workflow analysis. Although a limited number of samples can be extracted in one batch in these instruments, the turnaround time of the QMT assay (2.5 h) is significantly shorter than that of the RGQ assay (6 h) (Figure 1). Overall, these two assays provide reliable and comparable results.

## 5. Conclusions

The newly established fully automated real-time PCR assay enables the effective and early detection of CMV in various clinical specimens, particularly for respiratory tract specimens. It may also serve to provide accurate and timely information for effective clinical treatment to reduce the mortality rate of immunocompromised patients with CMV diseases.

## Figures and Tables

**Table 1:** Extraction conditions for the BD MAX instrument for different specimen types.

Test	Lysis Time (min)	Lysis Temperature (°C)	Sample Volume for Extraction (µL)	Wash Volume (µL)	Specimen Volume Added to SPT ^a^ (µL)
TM_CMV_STOOL	20	50	300	500	10
TM_CMV_CSF/VTM ^b^/URINE	9	62	700	500	200
TM_CMV_SERUM/PLASMA	10	37	700	500	200
TM_CMV_SPUTUM	20	50	700	500	200
TM_CMV_BAL ^c^	20	62	700	500	200

^a^ SPT, sample-processing tube. ^b^ VTM, viral transport medium. ^c^ BAL: bronchoalveolar lavage.

**Table 2:** The Performance of QMT assay with various clinical specimens.

Assayed	QMT Assay (*n* = 1067)		Detection Rate of QMT Assay [%(A/B) ^a^]	Invalid Rate of Internal Positive Control PCR [%(A/B) ^b^]
Specimen (Total Number Tested)	CMV Positive	CMV-Negative		
Plasma (426)	110	316 (6) ^#^	25.8 (110/426) *	1.4 (6/426)
Respiratory tract specimens (293)	127	166	43.3 (127/293)	
BAL ^c^ (167)	80	87 (14) ^#^	47.9 (80/167) *	8.4 (14/167)
Sputum (57)	29	28 (6) ^#^	50.9 (29/57)	10.5 (6/57)
Throat swab (36)	11	25 (1) ^#^	30.6 (11/36)	2.8 (1/36)
NP swab ^d^ (33)	7	26 (2) ^#^	21.2 (7/33)	6.1 (2/33)
Stool (127)	27	100 (32) ^#^	21.3 (27/127)	25.2 ** (32/127)
Urine (18)	5	13 (3) ^#^	27.8 (5/18)	16.7 (3/18)
Breast milk (5)	4	1	80 (4/5)	0 (0/5)
Vitreous humour (90)	17	73 (6) ^#^	18.9 (17/90)	6.7 (6/90)
Bone marrow (5)	1	4 (4) ^#^	20 (1/5)	80 ** (4/5)
CSF (101)	1	100 (4) ^#^	1.0 (1/101)	4.0 (4/101)
Lung tissue (2)	2	0	100 (2/2)	0 (0/2)
Total number	294	773 (78) ^#^	27.6 (294/1067)	7.3 (78/1067)

^a^ A/B, positive samples/all specimens tested. ^b^ A/B, invalid samples/all specimens tested. ^#^ specimens with negative result of internal positive control PCR. ^c^ BAL, bronchoalveolar lavage; ^d^ NP swab, nasopharyngeal swab. * indicated *p* < 0.05 among CMV DNA detection rate and ** indicated *p* < 0.05 among rate of invalid internal positive control by post hoc statistical analysis.

**Table 3:** CMV nucleic acid detected in simultaneous specimens of respiratory tract and plasma in patients with respiratory symptoms by QMT assay.

Co-morbidity Disease	Collected Specimens from Same Patients for QMT Assay			Detection Rate of QMT Assay		
	RTS^+^ and P^+^	RTS^+^ and P^−^	RTS^−^ and P^+^	RTS	*p*	*p* Value **
Transplant—10 ^#^ (10 ^@^)	6 (6)	4 (4)	0	100% (10/10) ^a^	60% (6/10)	<0.0001
Malignancy—32 (31)	20 (19)	12 (12)	0	100% (32/32)	62.5 (20/32)	<0.0001
Immunodeficiency—13 (11)	8 (8)	5 (3)	0	100% (13/13)	61.5 (8/13)	<0.0001
Diabetes mellitus, hypertension, cardiovascular disease—33 (32)	23 (22)	8 (8)	2 (2)	93.9 % (31/33)	78.1% (25/32)	0.0043
Other—6 (6)	2 (2)	3 (3)	1 (1)	83.3% (5/6)	50% (3/6)	<0.0001
Total episodes ^&^ (All patients)—94 (91)	59 (58)	32 (30)	3 (3)	93.6% (88/94)	65.9% (62/94)	<0.0001

** *p* value calculated by Fisher’s exact test. #: number of episodes; ^@^: number of cases; ^a^: positive episodes/total episodes. RTS: respiratory tract specimen; P: plasma specimen. ^&^ episode: an episode was defined as the period began with positive result of QMT assay for collected blood or respiratory specimens from same patients with respiratory symptoms and ended with two consecutive negative detection tests.

**Table 4:** Accuracy of the QMT assay as determined using College of American Pathologists Proficiency Testing (CAP-PT) specimens as external quality control specimens for qualitative and quantitative analysis.

Qualitative Analysis			
CAP specimen	QMT assay (log_10_ IU/mL)	In-house assay	CAP-PT report
18-ID1-03	Positive (6.15)	Positive	Positive
17-ID1-03	Negative	Negative	Negative
17-ID1-11	Positive (4.81)	Positive	Positive
16-ID1-03	Positive (6.36)	Positive	Positive
16-ID1-11	Positive (6.79)	Positive	Positive
**Quantitative Analysis (log_10_ IU/mL)**			
CAP specimen ^a^	QMT assay	Roche assay	CAP-PT mean per group
18-VLS-03	3.66	3.60	3.76
18-VLS-04	4.73	4.92	4.71
18-VLS-13	3.46	3.78	3.64
18-VLS-14	4.77	4.67	4.82
17-VLS-03	Negative	Negative	Negative
17-VLS-04	3.73	3.34	3.83
17-VLS-13 ^b^	3.49	3.95	3.78
17-VLS-14 ^b^	4.83	5.02	4.90
16-VLS-03	2.01	<2.14	2.25
16-VLS-04	3.63	3.17	3.35
16-VLS-13	Negative	Negative	Negative
16-VLS-14	3.39	3.27	3.43

CAP-PT specimens: ^a^ 2-fold dilution; ^b^ 10-fold dilution.

**Table 5:** Comparison of the performance of in-house, RGQ, and QMT assays for the analysis of frozen residual plasma specimens positive in Roche assay.

Viral Load (IU/mL)	In-House Assay		RGQ Assay		QMT Assay	
*n* = 60	Positive	Negative	Positive	Negative	Positive	Negative
<137 (*n* = 40)	2	38	18	22	26	14
137–500 (*n* = 10)	7	3	10	0	10	0
500–1000 (*n* = 10)	10	0	10	0	10	0

**Table 6:** Diagnostic performance of in-house, RGQ, and QMT assays with various clinical specimens.

*n* = 205	In-House Assay% (A/B) ^a^	RGQ Assay % (A/B) ^a^	QMT Assay% (A/B) ^a^
Diagnostic sensitivity ^b^	65.5 (72/110)	100 (110/110)	99.1 (109/110)
Diagnostic specificity ^b^	100 (95/95)	87.3 (83/95)	91.5 (87/95)
Positive predictive value ^b^	100 (72/72)	90.2 (110/122)	93.1 (109/117)
Negative predictive value ^b^	71.4 (95/133)	100 (83/83)	98.9 (87/88)

^a^ A/B, positive samples/all specimens tested. ^b^ Two positive or two negative results in these assays were considered as true positive and true negative testing, respectively.
